# Correction: Ozone inactivation of airborne influenza and lack of resistance of respiratory syncytial virus to aerosolization and sampling processes

**DOI:** 10.1371/journal.pone.0334842

**Published:** 2025-10-16

**Authors:** Marie-Eve Dubuis, Étienne Racine, Jonathan M. Vyskocil, Nathalie Turgeon, Christophe Tremblay, Espérance Mukawera, Guy Boivin, Nathalie Grandvaux, Caroline Duchaine

The following information is missing from the Funding statement: CD: Institut de recherche Robert-Sauvé en santé et sécurité du travail (IRSST 2017-0004).
