# Supplementary material for: Ozone inactivation of airborne influenza and lack of resistance of respiratory syncytial virus to aerosolization and sampling processes
Source: PLoS One. 2021 Jul 12;16(7):e0253022. doi: 10.1371/journal.pone.0253022 (PMC8274922; doi:10.1371/journal.pone.0253022)
Supplement: S1 File — (DOCX) [file pone.0253022.s001.docx]

# S1 File– Regression analyses

## 34% RH

For 34% humidity, the ozone concentration $C(t)$ decays exponentially with time $t$. We therefore performed a linear regression for the natural logarithm of ozone concentration (expressed in ppm) versus time (expressed in minutes):

$\ln C(t)= \alpha- \lambda t$,

where $\alpha$ is the intercept and $\lambda$ is the decay constant. Linear regression was performed in RStudio and the full results are:

**S1 Table: Estimated regression coefficients, standard errors, confidence intervals and p-values for the 34% RH regression analysis.**

| **Coefficient** | **Estimate** | **Standard error** | **95% confidence interval** | ***p*-value** |
| --- | --- | --- | --- | --- |
| $\alpha$ | 0.889320 | 0.065855 | 0.757976 – 1.02066 | < 2.0e-16 |
| $\lambda$ | 0.036039 | 0.001745 | 0.032559 – 0.039518 | < 2.0e-16 |

Goodness-of-fit was assessed with the multiple R-squared statistic:

$R^{2}= 0.8591$ .


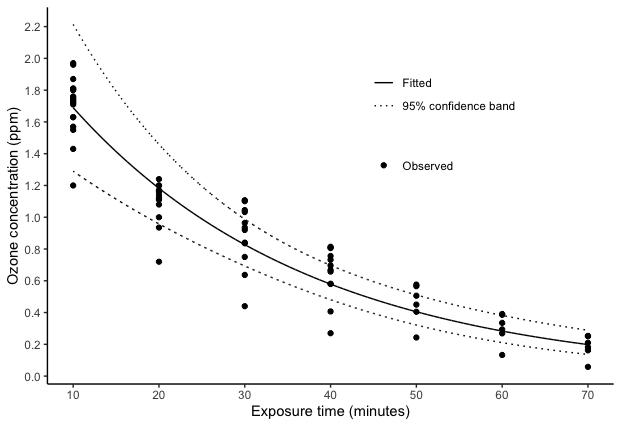


**S1 Fig: Regression of the ozone concentration at 34% RH for exposure times between 10 and 70 minutes.** Each reading is represented by a black circle (●). The full line represents the fitted linear regression and the dotted lines represent the 95% confidence bands.

## 76% RH

For 76% humidity, the ozone concentration $C(t)$ decays exponentially with time to the power 0.125, i.e. $t^{1/8}$. We therefore performed a linear regression for the natural logarithm of ozone concentration (expressed in ppm) versus transformed time $\tau= t^{1/8}$ (expressed in min^1/8^):

$\ln C(t)= \alpha- \lambda\tau$,

where $\alpha$ is the intercept and $\lambda$ is the decay constant with respect to transformed time. Linear regression was performed in RStudio and the full results are:

**S2 Table: Estimated regression coefficients, standard errors, confidence intervals and p-values for the 76% RH regression analysis.**

| **Coefficient** | **Estimate** | **Standard error** | **95% confidence interval** | ***p*-value** |
| --- | --- | --- | --- | --- |
| $\alpha$ | 12.2100 | 0.7911 | 10.6322 – 13.7878 | < 2.0e-16 |
| $\lambda$ | 9.9035 | 0.5215 | 8.8633 – 10.9437 | < 2.0e-16 |

Goodness-of-fit was assessed with the multiple R-squared statistic:

$R^{2}= 0.8374$ .


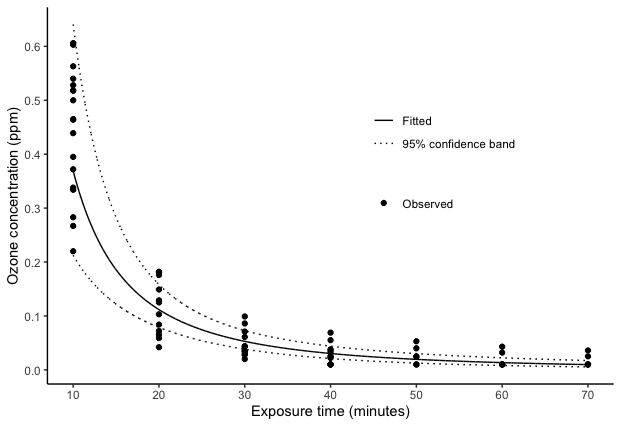


**S2 Fig: Regression of the ozone concentration at 76% RH for exposure times between 10 and 70 minutes.** Each reading is represented by a black circle (●). The full line represents the fitted linear regression and the dotted lines represent the 95% confidence bands.
